# Intelligent Perception, Decision-Making and Actuation Technologies for Precision Agrochemical Spraying: Current Advances and Future Perspectives

**DOI:** 10.3390/s26175403

**Published:** 2026-08-26

**Authors:** Qi Song, Fu Zhang, Zhen Ma, Bingbo Cui, Cundeng Wang

**Affiliations:** 1 School of Agricultural Engineering, Jiangsu University, Zhenjiang 212013, China; songqi@ujs.edu.cn (Q.S.);; 2 School of Instrument Science and Engineering, Southeast University, Nanjing 210096, China; 230239006@seu.edu.cn

**Keywords:** precision agriculture, intelligent spraying system, target recognition, atomization characteristics

## Abstract

**Highlights:**

This review examines intelligent spraying systems across perception, actuation, and multi-platform integration in agriculture. Compares 2D field and 3D orchard spraying systems in sensing, control, and fluid dynamics performance.

**What are the main findings?**

- Sensing accuracy and actuation performance are affected by environmental variability and dynamic delays in the system.
- Fluid dynamics, droplet formation, and canopy structure significantly influence deposition uniformity and drift behaviors.

**What are the implications of the main findings?**

- Integrated CPS and digital twin frameworks are needed to improve coordination between sensing and actuation systems.
- Future systems should combine multimodal sensing and feedforward control to reduce drift and improve efficiency.

**Abstract:**

This review summarizes recent advances in intelligent spraying systems for precision agriculture. It focuses on the three interconnected components of perception, decision-making, and actuation, and compares the sensing characteristics of 2D planar field crops and three-dimensional orchard canopies. Particular emphasis is placed on evaluating the target recognition capabilities and physical limitations of machine vision, Light Detection and Ranging (LiDAR), ultrasound, and active fluorescence spectroscopy under complex field conditions. At the actuation level, the dynamic response characteristics of Pulse-Width Modulation (PWM), proportional control valves, and adaptive control algorithms are critically reviewed, revealing the influence mechanism of hydraulic transient phenomena, including water hammer effects induced by high-frequency valve switching, on droplet size distribution (DSD). The review further discusses the coupling effects between Unmanned Aerial Vehicle (UAV) airflow fields, Unmanned Ground Vehicle (UGV) motion disturbances, and spray deposition performance, and summarizes reported improvements in pesticide reduction, water conservation, and drift mitigation. Finally, the potential of cyber–physical systems (CPS) and digital twin-based frameworks for developing adaptive and closed-loop intelligent spraying systems is discussed to provide insights into future all-weather and autonomous agricultural operations.

## 1. Introduction

### 1.1. Background and Development of Intelligent Spraying Systems

With the continued growth of the global population and the increasing demand for food security, modern agriculture is facing the dual challenge of increasing crop yields and reducing ecological impact. In this context, precision agriculture technology, especially intelligent spraying systems, has gradually become a core research direction in the field of agricultural engineering. Conventional uniform spraying not only leads to serious waste of pesticides and fertilizers, but also causes soil and water pollution as well as ecological damage in non-target areas. Therefore, it is particularly crucial to develop an intelligent spraying system that can perceive crop canopy characteristics in real time, identify diseases, pests, and weeds, and dynamically adjust spray rates. At the underlying physical mechanism level of spraying technology, the collision dynamics, interface wettability, and atomization characteristics of droplets have a decisive impact on the final deposition effect. Wang et al. pointed out that the complex microstructure of crop leaf surfaces determines their hydrophilic or hydrophobic properties. By adjusting surfactants and reducing droplet size, the precise deposition behavior of pesticides at complex interfaces can be significantly improved [1]. In order to analyze this process, computational fluid dynamics (CFD) and multiphase flow models have been widely applied in the study of atomization mechanisms. For example, Chen et al. successfully simulated the complex fluid dynamics process of generating large droplets in an internal mixed airflow-assisted nozzle using a fluid volume-to-discrete-phase model conversion method, providing high-fidelity predictive support for the optimization of nozzle geometric parameters [2]. Jiang et al. employed large eddy simulation (LES) coupled with level-set and volume-of-fluid (VOF) methods to reveal the axis switching behavior of non-circular nozzle jets. They found that the diffusion angle of square nozzles was 37% larger than that of circular nozzles, providing a theoretical basis for nozzle design in low-pressure sprinkler irrigation systems [3]. At a more microscopic level, Jiang et al. further demonstrated that the above numerical simulation methods have an error of less than 10% in extracting jet rupture characteristics by combining the study of three-dimensional flow field rupture characteristics [4].

In addition to optimizing the fluid dynamics characteristics, the mechanical stability and automation level of spraying equipment are also key factors affecting the quality of spraying. Do Nascimento e Silva et al. proposed a mathematical model based on rheological magnetic damping to solve the dynamic vibration problem of orchard tower spray, successfully reducing the trailer vibration amplitude by 23.68%, significantly improving the uniformity of spray distribution and the safety of equipment operation [5]. In terms of system integration and validation, Cutini et al. developed a SimAgri collaborative simulation system based on virtual reality, which provides a safe and efficient virtual laboratory environment for fine-tuning and testing precision agricultural operation logic by integrating automatic navigation, geolocation and ISOBUS interface [6]. In recent years, the widespread application of drones and ground robots in the field of plant protection has further promoted the integration of perception and control technology. Researchers are constantly exploring new modeling and algorithm strategies to address the issue of control accuracy in spraying systems. Choudhury et al. constructed a high-fidelity prediction model using artificial neural networks, accurately quantifying the reverse effect of surface roughness on the maximum spreading factor of impact droplets [7]. Hu et al. designed a multi-fluid atomizer combining an axial nozzle and a swirl mixing chamber, which can generate ozone droplets with an average spatial resolution of 102 μm and a concentration of up to 3.73 mg/L at 0.6 MPa, providing innovative hardware support for green plant protection [8]. At the system-level application, Salas et al. validated the effectiveness of a variable spraying system equipped with ultrasonic sensors in apple orchards using the European OPTIMA project. The results showed that the system could reduce pesticide use by up to 35% while ensuring canopy deposition coverage [9].

### 1.2. Challenges in Different Agricultural Spraying Paradigms

**Figure 1:**
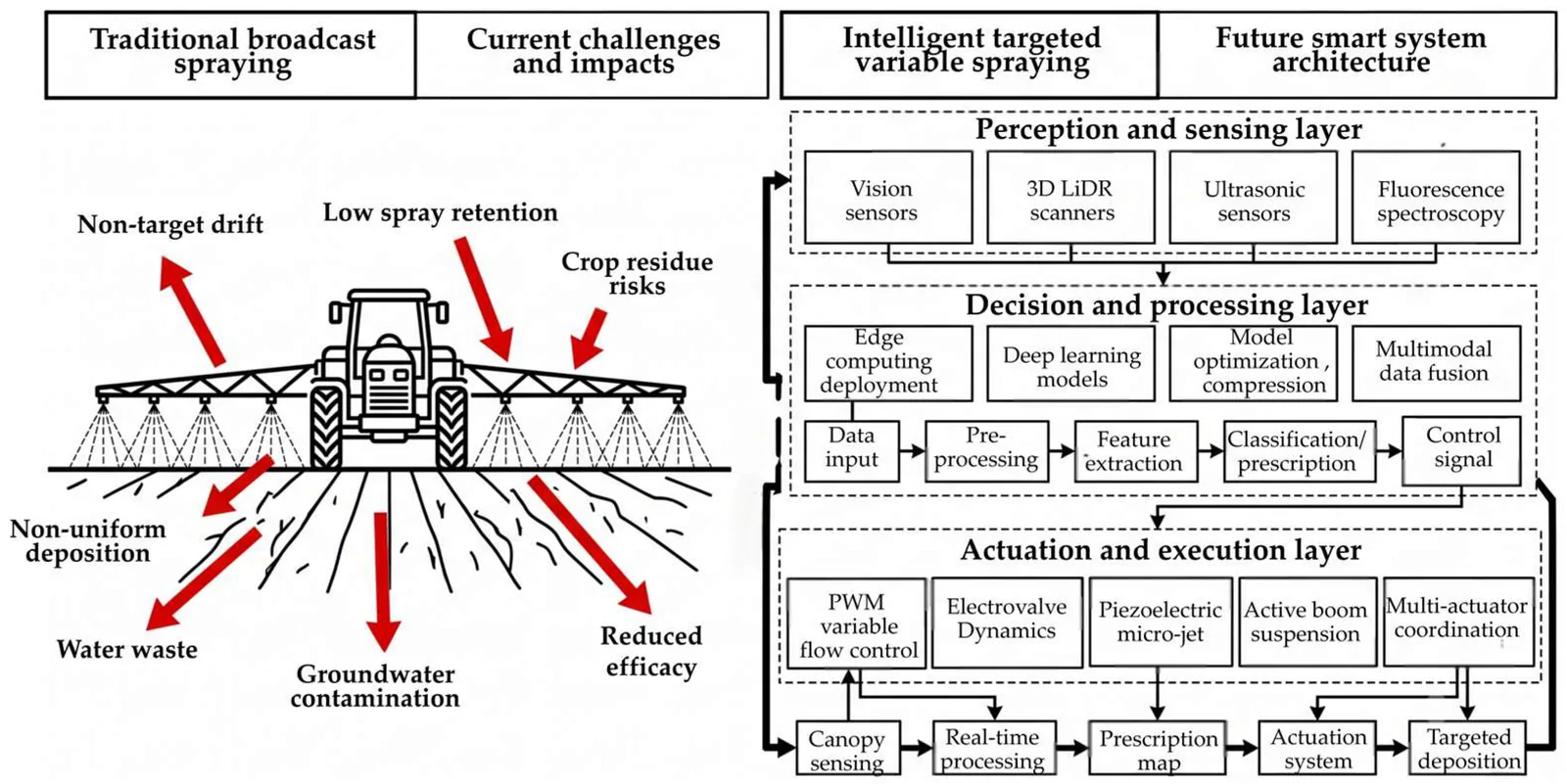
Comparison of traditional methods and intelligent spraying systems.

Figure 1 illustrates the structural and operational differences between traditional spraying methods and modern intelligent spraying systems. The precision flat field operation typically employs the large-span boom spraying method. The core engineering issues during the operation process focus on the high-frequency response performance of the control system when the machinery is running at high speed, as well as the pipeline water hammer effect induced by frequent valve opening and closing. The three-dimensional hierarchical operation typically employs air-assisted orchard spraying and aerial plant protection, and the corresponding core problem is the insufficient penetration ability of liquid pesticide into the multi-layer, complex leaf canopy of fruit trees, as well as the disordered drift of gas–liquid multiphase flow under the interference of the environmental wind field. Therefore, the subsequent review chapters of this article will focus on sorting and evaluating these two different agricultural operation paradigms.

### 1.3. Literature Identification and Review Methodology

To summarize the current development status and future trends of precision agriculture intelligent spraying systems, this review provides a comprehensive analysis of related studies based on technological evolution and system functional relationships. The identification, classification, and evaluation of the literature mainly include the following three aspects.

(1) Scope of the literature search and keyword selection

This review conducts the literature research on the three key components of intelligent spraying systems, namely perception, decision-making, and actuation. The main search sources include international databases such as Web of Science and Scopus. Keyword combinations were selected according to the research objectives, including “precision spraying”, “intelligent spraying system”, “multimodal sensing”, “machine vision”, “LiDAR”, “variable-rate application”, “spray control”, “UAV”, and “UGV”, covering related research directions such as target recognition, multi-source perception, variable-rate spraying, unmanned operation platforms, and intelligent control.

(2) Principles of the literature classification and selection

The classification and selection of literature were mainly based on research relevance and consistency with the technical framework of this review, with a focus on studies that directly support precision agricultural spraying tasks. According to technical functions, related studies were categorized into three groups:

Perception technologies for identifying crops, weeds, pests, and diseases. 

Spray dynamics, variable-rate control, and actuator optimization technologies. 

System integration and autonomous operation technologies for UAV and ground-based autonomous platforms. 

Studies that mainly focused on industrial spraying, fuel injection, or non-agricultural fluid processing applications were considered only as fundamental theoretical references and were not included in the core technical analysis.

(3) The literature evaluation and technical analysis methods

This review evaluates relevant studies mainly from the perspectives of technological innovation, experimental validation, engineering maturity, and adaptability to agricultural environments. Among these, perception technologies are evaluated based on target recognition capability, multi-source information fusion, and environmental robustness. Actuation technologies are analyzed in terms of dynamic response, flow regulation performance, and operational stability. System integration studies are evaluated according to the information exchange and closed-loop control capabilities among perception, decision-making, and actuation modules.

### 1.4. Scope and Contributions of This Review

This review provides an overview of the complete technology chain of intelligent spraying systems, with particular emphasis on the coupling relationships among multi-source perception, intelligent decision-making, and adaptive actuation. The main contributions of this review are as follows:

(1) Building a closed-loop technical analysis framework for intelligent spraying systems

Unlike existing reviews that focus on individual technology modules, this review analyzes the development of key technologies from the perspective of a perception–decision-making–actuation cyber–physical closed-loop framework, revealing the data interactions and functional coupling relationships among different modules.

(2) Comparison of technical requirements under different agricultural operation modes

This review distinguishes between two typical scenarios, namely 2D planar field crop spraying and three-dimensional orchard canopy spraying, and analyzes their key challenges in target perception, spatial information acquisition, droplet transport, and operational control.

(3) Summary of development bottlenecks and future directions of intelligent spraying systems

This review discusses major challenges, including multimodal perception uncertainty, dynamic response limitations of actuation systems, adaptability to complex agricultural environments, and closed-loop intelligent control. Furthermore, the potential applications of artificial intelligence and cyber–physical systems (CPS) in next-generation autonomous spraying equipment are analyzed.

## 2. Intelligent Perception for Crop Target and Canopy Characterization

The intelligent variable pesticide application system is divided into three levels in a logical architecture: perception, decision-making, and execution. The perception layer relies on multimodal sensors to extract target features, and the decision-making layer generates spatial prescription instructions based on these features. The execution layer ultimately drives the physical hardware to complete the chemical application. This section focuses on the technological evolution of the perception layer and strictly distinguishes the physical logic, technical pain points, and application boundaries between 2D field flat crops and 3D orchard stereo crops in object detection tasks.

### 2.1. Vision-Based Perception and Deep Learning Technologies

#### 2.1.1. 2D Weed Detection and Edge-AI Deployment in Open-Field Spraying

In the precise application of 2D field crops, the boom sprayer usually uses a machine vision system with a vertical downward perspective to extract the row spacing of crops or identify specific weeds. The core engineering challenge of this scenario is how to achieve low latency and high-precision inference in the high-speed driving state of the tractor. The You Only Look Once series of algorithms has become the mainstream solution for edge computing device deployment because it offers a good balance between feature extraction speed and bounding box regression accuracy. Related studies have shown that under controlled lighting or specific constructed datasets, optimized deep learning models can achieve extremely high classification accuracy for weed targets, verifying the theoretical feasibility of integrating low-cost cameras with single-board computers for autonomous weed control [10,11].

However, when the visual perception system moves from greenhouses to complex unstructured field environments, its model generalization ability faces a severe test. Under natural field conditions, the real-time detection accuracy of visual models often drops sharply due to the interference of strong light alternation, mixed background soil spectra, and overlapping crop growth in multiple seasons, leading to a significant decline in the targeted hit rate of boom sprayers [12,13]. In addition, the robustness of different classification networks in actual operations varies significantly. When the driving speed of the boom sprayer exceeds 3 km/h, due to motion blur and hardware sampling frequency restrictions, the target capture rate of the visual system will show systematic degradation [14]. Existing studies indicate that increasing network depth alone cannot overcome the generalization bottleneck. The optimization of perception systems for planar field crops must shift towards building a standard dataset containing multi-source environmental noise and synchronously upgrading the spatial resolution of visual hardware.

#### 2.1.2. Estimation of 3D Canopy Structure and Foliage Density

**Figure 2:**
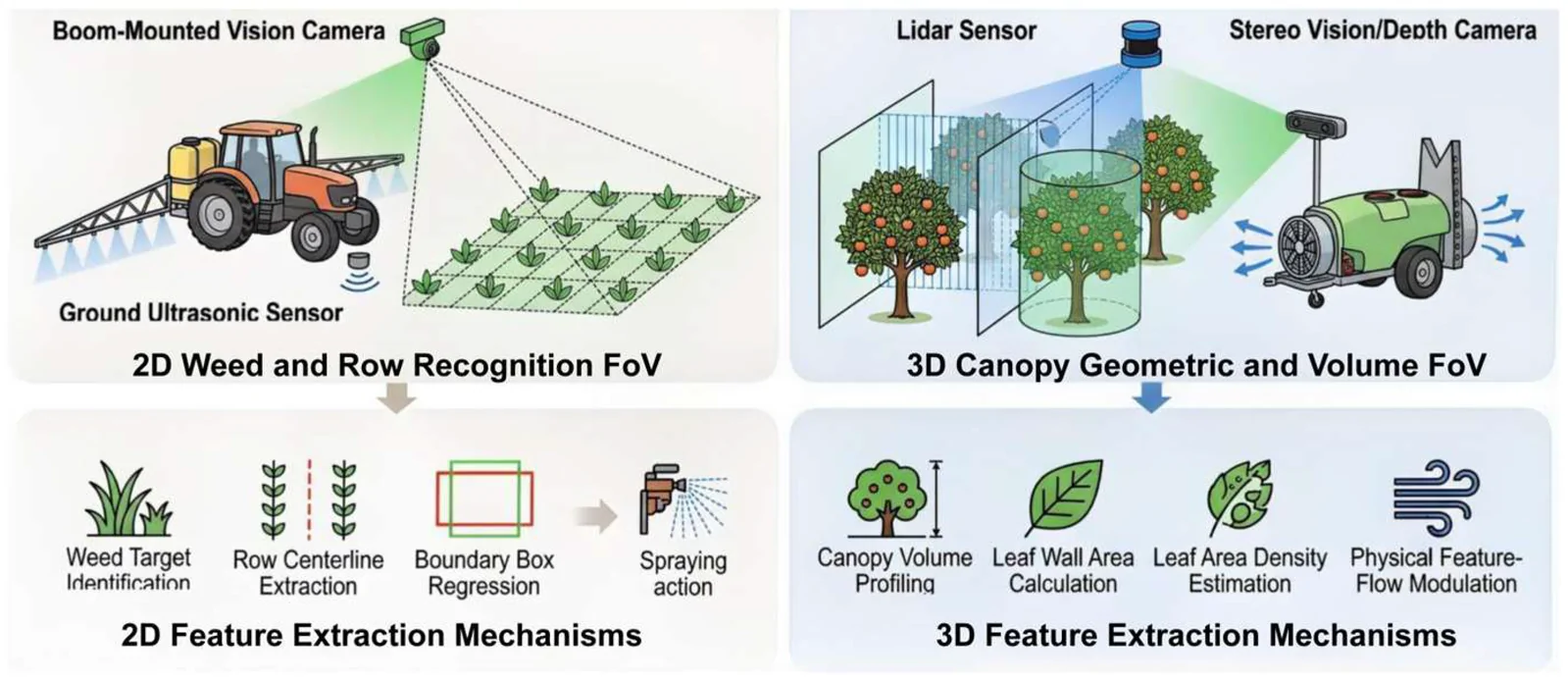
Schematic of 2D and 3D canopy perception and feature extraction for spraying.

Different from the downward perspective of planar field-crop scenarios, the application operation of 3D crops, such as high-density orchards and vineyards, mainly relies on aerial spraying. Its visual perception system adopts a lateral viewing perspective to estimate canopy foliage density and geometric depth [15]. Due to the highly anisotropic porosity of the three-dimensional canopy structure and the severe self-occlusion of branches and leaves, traditional 2D object bounding box detection models find it difficult to directly characterize the spatial canopy porosity of the 3D canopy. For 3D canopy, the research focus is gradually shifting towards using stereo vision or depth imaging modules to track the outer contour of fruit trees and invert leaf area density. Partial research based on commercial depth cameras has made some progress in outdoor fruit tree contour tracking, but due to optical ranging principles, such sensors are extremely sensitive to environmental lighting [16,17]. In long-distance and large-scale spraying regulation, strong direct sunlight can easily cause point cloud voids and geometric accuracy degradation. For 3D crops, the core pain point of visual perception is no longer the qualitative recognition of a single biological target, but how to overcome the high-frequency dynamic deformation of leaves caused by strong wind-assisted airflow and stably extract the effective porosity of the canopy in complex agricultural backgrounds. The schematic diagram of dual-mode canopy perception and feature extraction is shown in Figure 2.

#### 2.1.3. Lightweight Deep Learning and Low-Latency Edge Inference

To meet the demanding real-time requirements of mobile agricultural equipment, model optimization, compression and hardware acceleration technologies are indispensable. By quantizing the model in an integer data format and optimizing the network backbone architecture, inference time can be effectively controlled at the millisecond level, while significantly compressing the model volume to adapt to resource-limited field positioning needs [18,19]. At the micro visual level, the combination of high-speed camera technology and data-driven frameworks provides direct observation methods for droplet trajectory tracking and collision dynamics evaluation [20,21,22].

Crucially, variations in the physicochemical properties of plant leaf surfaces constitute a key physical variable that passive machine vision systems typically overlook. The existing review analysis indicates that relying solely on the geometric features of appearance for pesticide application decisions often leads to a significant deviation in the sedimentation efficiency of the drug at complex interfaces from expectations. By introducing physical intervention methods such as electric field assistance and interface modification, the dynamic contact angle and wet diffusion behavior of droplets can be significantly changed, effectively suppressing the coffee-ring effect and improving interface adhesion [23,24,25]. This indicates that future intelligent visual spray systems must go beyond a single phenotypic perception category, integrate the physical and chemical parameters of the leaf surface into the decision-making framework, and promote the evolution of the system toward multi-physical-field collaborative control. The spatiotemporal characteristics and algorithm performance of different vision-based sensing paradigms are summarized in Table 1.

### 2.2. Active Remote Sensing for 3D Canopy Quantification

#### 2.2.1. LiDAR-Based Estimation of Tree Row Volume and Leaf Wall Area

Compared to passive visual systems that are susceptible to light interference, Light Detection and Ranging (LiDAR) exhibits excellent measurement stability in quantifying the spatial geometry of 3D crops. In precise variable pesticide application in orchards, Tree Row Volume (TRV) and Leaf Wall Area (LWA) are the primary geometric descriptors for generating three-dimensional pesticide prescription maps. For microscopic spray modeling, Brentjes et al. proposed a new method to estimate the droplet charge in the numerical simulation of conductively and inductively charged spray. By balancing the global electric field, the high computational cost caused by local high resolution was avoided [33]. Herkins et al. used CFD models to predict the settling and drift of electrostatic spraying. Under windless and 2.24 m/s wind speeds, the average relative errors of the model for canopy deposition were 40.3% and 58.8%, respectively [34]. Kooij et al. quantified the self-charging phenomenon of spray, proposed a simple proportional model, integrated the flow current data across four orders of magnitude under different nozzle sizes and flow rates, and revealed the variation pattern of charge effect on spray plume shape [35]. 

**Figure 3:**
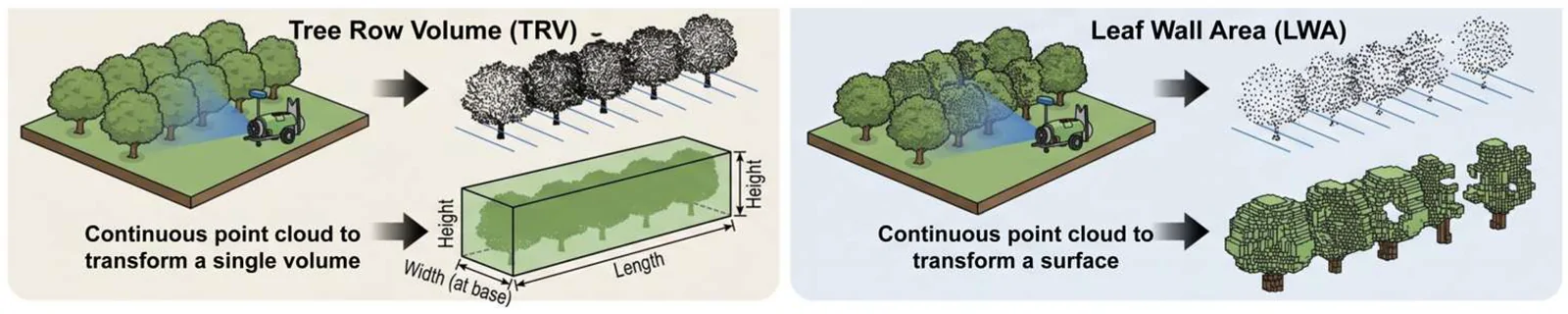
Schematic comparison of TRV and LWA canopy geometric modeling methods.

By comparing different geometric dose models horizontally, it can be found that the TRV model can better meet the requirements of full canopy coverage when dealing with dense and uniform canopies. However, when facing a canopy that is in the pruning period or naturally growing sparsely, simple volume fitting often leads to serious risks of liquid penetration and drift. At this time, the LWA model based on the optimal coverage area shows higher rationality of liquid deposition [36,37,38]. Although LiDAR provides a high-precision 3D decision-making foundation, its scanning blind spots and high system costs remain the main obstacles to its widespread adoption. A visual comparison of TRV and LWA canopy geometric modeling approaches is presented in Figure 3.

#### 2.2.2. Point-Cloud Processing, Geometric Errors, and Structural Parameter Extraction

With the development of solid-state LiDAR technology, agricultural 3D perception is evolving toward miniaturization and high-frequency data acquisition capability. LiDAR point clouds can effectively characterize the spatial structure parameters of crop canopies and are widely used in precision spraying scenarios. Compared to ranging accuracy, the propagation of errors during point cloud denoising, segmentation, and parameter extraction is the core bottleneck that restricts their stable application in the field.

In terms of point cloud segmentation and structure extraction, uneven point cloud density, leaf occlusion, and plant overlap are the main issues affecting the accuracy of canopy reconstruction. Traditional methods based on geometric rules are characterized by high computational efficiency and strong interpretability. Lin et al. established a corn single-plant point cloud segmentation model based on DBSCAN, achieving an overall accuracy of 0.94–0.98 on different test plants, and further achieving hierarchical recognition of organs such as ears, stems, and leaves [39]. Zeng et al. used 3D LiDAR geometric features to carry out semantic segmentation of trellis wires, support rods, and tree trunks in apple orchards, and constructed canopy density and depth distribution maps to provide structural information for precise fruit tree pruning and variable spraying [40]. However, this type of method relies on prior knowledge of crop morphology and has limited generalization ability under different varieties, growth stages, and complex occlusion conditions.

In recent years, deep learning has driven the transition of point cloud processing from rule-based to data-driven approaches. Jayakumari et al. proposed CropPointNet, which achieved object-level classification of vegetables based on LiDAR point clouds, with classification accuracy exceeding 81% for different crops [41]. Hao et al. used PointSegAt to achieve cotton stem and leaf segmentation and overlapping leaf recognition, with a segmentation accuracy of 0.995 and mean intersection over union (mIoU) of 0.924, and further extracted phenotypic parameters such as plant height, leaf area, and canopy area [42]. Luo et al. improved the PointResNet model by integrating spatial position, color, and normal vector information to achieve point cloud segmentation of grape clusters, stems, and leaves, with an overall accuracy of 96.5% [43]. The above research indicates that the development focus of point cloud algorithms has shifted from single geometric segmentation to multi-feature fusion to enhance target recognition capabilities in complex canopy environments.

Further, the research has gradually expanded from point cloud segmentation to the extraction of structural parameters for spray decision-making. Zhao et al. used solid-state LiDAR to conduct dynamic monitoring of maize plant height, achieving high accuracy at multiple growth stages. The root mean square error (RMSE) was 1.27 cm, MAPE was about 0.77%, and the highest R^2^ reached 0.99 [44]. Luo et al. applied LiDAR to the Unmanned Aerial Vehicle (UAV) variable spraying system, estimating the cotton canopy volume through SMRF filtering, alpha shape, convex hull, voxel, and other methods, among which the alpha shape method showed the highest consistency with manual measurements, and further drove the dynamic adjustment of the spray rate, achieving a 43.37% reduction in the spray volume [45]. This study demonstrates that the core value of LiDAR lies not only in improving 3D measurement accuracy, but also in establishing a direct mapping relationship between canopy structure perception, parameter quantification, and variable spraying.

At the same time, point cloud processing is developing towards semantic environmental understanding. Sun et al. proposed Canopy3D-Net, which improved the point cloud segmentation performance of fruit tree canopies through height-aware sampling, multi-feature fusion, and mixed loss optimization. It achieved a mIoU of 0.849 and an OA of 0.938 on the citrus dataset [46]. Research has shown that future agricultural 3D perception systems need to evolve from simply obtaining geometric information to a semantic 3D model that simultaneously describes “target type, spatial location, structural scale, and spraying requirements”.

However, solid-state LiDAR still faces problems such as sparse point clouds, occlusion, motion distortion, and environmental noise in actual high-speed operating environments. Vehicle vibration, crop oscillation, and platform movement can cause temporal inconsistencies in point clouds, resulting in fluctuations in structural parameters. The differences in laser divergence and target reflectance in remote measurement can also introduce edge localization errors. Therefore, future research needs to further shift from simple evaluation of segmentation accuracy to geometric error modeling, spatiotemporal registration, and uncertainty propagation analysis, and clarify the propagation mechanism of 3D perception errors to canopy parameters and spraying decisions.

In particular, in long-distance and large-scale spraying scenarios, laser pulse divergence and unstructured environmental noise may further degrade geometric ranging and canopy reconstruction accuracy. Accordingly, future algorithm development should not only focus on improving the accuracy of 3D point cloud reconstruction, but should also strengthen error compensation and noise filtering mechanisms to ensure that the extracted spatial information provides reliable agronomic guidance for spraying decisions. The technical parameters and application characteristics of mainstream 3D canopy quantitative sensing technologies are analyzed in Table 2.

### 2.3. Alternative Sensing Modalities and Field Applications

#### 2.3.1. Ultrasonic and Active Fluorescence Sensing

Unlike LiDAR, which focuses on obtaining canopy geometry, ultrasound and active fluorescence sensing can supplement crop status information through acoustic and biophysical responses, respectively. Ultrasound can perform distance and structural detection, while active fluorescence supports crop identification, physiological monitoring, and spray deposition evaluation, thereby improving multidimensional perception in agricultural spraying systems. Ultrasonic sensors are often used to detect the height of the spray bar and the distance between the canopy and the spray bar in farmland. Wu et al. constructed an ultrasonic detection model for wheat throughout its entire growth period, with a detection error of less than 50 mm within the typical operating range [56]. This model has the advantages of low cost and rapid response capability, and can meet the requirements of closed-loop spray boom height control. However, ultrasound is susceptible to interference from field environmental conditions and has limited spatial resolution, making it unable to replace LiDAR for 3D canopy reconstruction. Active fluorescence sensing has further expanded the dimensions of precision agriculture perception. Chlorophyll fluorescence can reflect changes in the plant photosynthetic system and provide information on physiological states that are difficult to obtain through traditional red–green–blue (RGB) vision. Quan et al. used fluorescence imaging to analyze the response of weeds to mechanical and chemical stress, and found that fluorescence parameters such as Fv/Fm, ETR, and NPQ can effectively reflect different stress states, providing a basis for evaluating spraying effectiveness [57].

Active spectral and fluorescence sensing technologies achieve high-precision target identification through the biophysical response of crops. Raja et al. proposed a concept of crop signal recognition. Combining machine vision and precision micro-spraying, at a driving speed of 3.2 km/h, the system could process crop images within 80 ms over an area of 120 mm × 180 mm, and the weed spraying accuracy in lettuce clusters was up to 98% [58]. The low-cost open-source fallow weed detection device OWL developed by Coleman et al. achieved an average detection accuracy of 79% and a recall rate of 52% in multiple test fields using color algorithms, demonstrating the application prospects of low-cost optical instruments in weed management [59]. Wang et al. further compared the performance of reflectance and fluorescence data in vegetation detection, confirming that fluorescence excited by blue and red light-emitting diodes is an effective attribute for distinguishing vegetation from non-vegetation. By modulating the light source with a sine signal, this low-cost sensor effectively resists the interference of ambient light and achieves 100% detection accuracy in both dark and outdoor environments [60].

In crop–weed recognition, active fluorescence enhances the difference between the target and background through active excitation. Zhang et al. used fluorescent markers combined with LED imaging systems to achieve automatic differentiation between tomato plants and weeds [61]. Jiang et al. further found that after appropriate tracer treatment, the fluorescence signal of crop tissue can reach about 10 times that of adjacent weeds, effectively improving the reliability of identification [62]. Compared to visual methods that rely on morphological features, this strategy can reduce misidentification caused by complex backgrounds and crop similarity. In addition, fluorescence technology can also be used for spray deposition detection and spraying quality evaluation. Yu et al. used Rhodamine-B fluorescence tracing combined with water-sensitive paper to establish a deposition correction method, which improved the consistency of deposition evaluation under different sampling conditions [63]. Lee et al. compared quantitative dye analysis and image analysis methods, and verified the application potential of fluorescence tracers in UAV spray deposition evaluation [64]. However, the fluorescence signal only reflects the spatial distribution of the tracer and cannot fully represent the actual spraying performance. Godyń et al. found that UAV spraying had problems, including insufficient canopy deposition and uneven deposition between the upper and lower canopy layers in the orchard, and that an increase in flight speed would lead to increased spray drift [65]. Yao et al. further pointed out that pre-wetting treatment and leaf inclination angle will significantly affect the UAV spray deposition effect [66]; Khankandi et al. showed that the rotor downwash airflow and ambient wind field can alter the droplet size and spatial distribution [67]. Overall, ultrasound and active fluorescence sensing have propelled precision spraying perception from single geometric detection to multidimensional information acquisition. In the future, it is necessary to improve signal stability and anti-interference ability, and integrate these sensors with LiDAR and machine vision to build a multimodal perception system.

#### 2.3.2. Ultrasonic Multi-Echo Sensing

Ultrasonic sensors are widely used to measure crop height and canopy thickness due to their low cost and complete insensitivity to ambient light. The analysis of the echo interval time and peak characteristics of ultrasound has proven useful for estimating leaf area density inside the canopy, with low relative errors in both indoor simulations and outdoor physical tree tests [68,69]. Although ultrasonic technology is inferior to LiDAR in terms of spatial resolution, it offers significant advantages in maintaining low maintenance costs for adaptive spray boom height control and inter-row distance management in orchards.

## 3. Dynamic Actuation and Flow Control Systems for Precision Spraying

Accurate crop canopy perception requires stable and reliable actuation systems for implementation. The modern variable pesticide flow control system can be logically divided into three levels: control principle, closed-loop control algorithm, and field condition compensation. This section will elaborate on the dynamic response mechanism and variable control logic of the above flow regulation system, and focus on analyzing the essential differences between 2D plane crop spray rod spray and 3D crop air spray in the bottom layer implementation technology and engineering pain points.

### 3.1. PWM-Based Variable-Rate Flow Control for Boom Sprayers

#### 3.1.1. Transient Dynamics of High-Frequency Solenoid Valves

Pulse-Width Modulation (PWM) technology is the core driving method for achieving local nozzle flow fine-tuning in the precise targeted application of 2D field flat crops. This technology achieves precise flow regulation by controlling the opening and closing time ratio, i.e., duty cycle, of high-frequency solenoid valves. However, it faces flow stability challenges in intermittent dynamic processes.

To quantify this dynamic behavior, Pokharel et al. established a mathematical model of nozzle pressure based on upstream pressure and control signals, pointing out that the damping ratio and natural frequency are highly dependent on the orifice size, and improper aperture can significantly prolong the pressure establishment time [70]. The core pain point caused by these transient dynamics is that high-frequency opening and closing can easily trigger strong water hammer effects in flexible pipelines. For the overall design of the system, Li et al. developed a profiling spray suitable for the dwarfing and dense planting mode, and achieved an adhesion rate of nearly 78.97% under a specific spraying distance and pressure [71]. Yan et al. built a real-time variable control system for multiple nozzles based on laser sensor guidance, and achieved 29.3–51.4% spray volume reduction [72] through space early opening and late closing control. In terms of predicting microscale atomization characteristics, Chen et al. used dimensional analysis to model the droplet size and spreading parameters of rotating cup atomization, successfully controlling the relative prediction error within a reasonable range [73]. In 2D high-speed spray boom operations, relying solely on macroscopic upstream pressure stabilization mechanisms is clearly insufficient to eliminate the underlying turbulence of microscopic flow fields.

#### 3.1.2. Hydraulic Oscillations and Their Effects on Droplet Deposition

Transient pressure fluctuations and hydraulic oscillations are common engineering challenges faced by multi-nozzle array systems. The sixteen-nozzle robot intelligent spraying platform designed by Vijayakumar et al. maintains the set pressure by simultaneously adjusting the duty cycle of the water pump and the proportional control valve, achieving fast establishment time and limited overshoot under step input [74]. However, this hydraulic oscillation not only acts on the inside of the pipeline, but also directly changes the microscopic fluid dynamics characteristics when droplets collide with solid surfaces.

The coupling calculation of multiple physical fields and the analysis of droplet deposition dynamics confirm that transient oscillations can alter the Weber number, thereby inducing droplet splashing, fragmentation, or the generation of secondary droplets. Wang et al. confirmed through numerical simulations that an external electric field can increase the critical velocity of droplet splashing and fragmentation, thereby promoting the spreading and fusion of droplets and liquid films [75]. Liu et al. conducted an in-depth analysis of the regression dynamics of droplet deposition on smooth surfaces and found that when the Weber number is in a specific range, the central jet will be pinched off to form a secondary droplet, and the capillary wave will retreat at a specific characteristic velocity [76]. To suppress adverse splashing caused by collisions, the introduction of dual synthetic jet technology or the design of rough surfaces with radial patterns can effectively limit the maximum stretching length and reduce the generation of satellite droplets [77,78]. Luo et al. proposed, based on a mass-spring model, that increasing the Weber number or decreasing the surface cylinder diameter can enhance anisotropy and effectively shorten the contact time of droplets [79]. Ding et al. revealed the direct influence of the shock wave generated by the entrained air flow on the spray width in the cross-impact spray through numerical simulation [80]. However, in actual 2D field operations, system design often considers water pumps and valves as independent linear physical domains and lacks joint adaptive compensation for the micro impact characteristics of nozzles and pipeline reflux pressure. With the increase in operating speed, the deviation of droplet deposition caused by transient oscillation will become increasingly severe.

#### 3.1.3. Effects of Duty Cycle on Droplet Size Distribution

**Figure 4:**
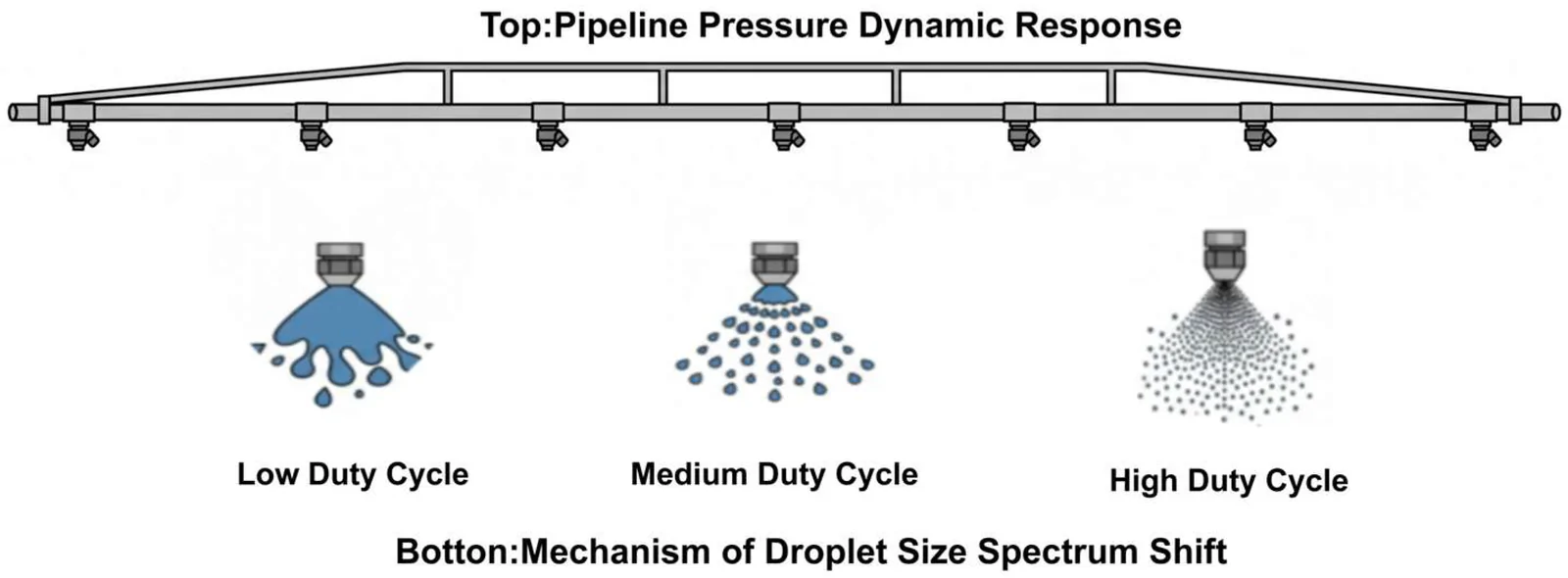
Schematic of droplet atomization characteristics at various PWM duty cycles.

The variations in droplet atomization characteristics under different PWM duty cycles are visualized in Figure 4. Another core technical pain point of PWM drive is that the dynamic change in flow fundamentally changes the energy input and physical fragmentation process of the spray, resulting in a significant nonlinear shift in the droplet size spectrum and spatial divergence angle under different duty cycles. The literature analysis shows that a sudden drop in pressure at extremely low duty cycles can cause fog droplets to thicken due to insufficient kinetic energy, resulting in a decrease in target coverage; at the moment of pressure rebound, small droplets are easily generated, exacerbating the risk of secondary drift [81,82]. Mhatre et al. studied the stability of the motion of settled charged droplets towards the substrate and proposed that the critical charge limit is determined by both the falling distance and the Bond number [83]. In terms of the stopping mechanism of moving droplets, Xu et al. elucidated the law that the airflow velocity required to control the droplet to stop moving with an air-blowing nozzle increases with the increase in droplet movement velocity and size [84]. The simulation results of Gultekin et al. showed that changes in the number and horizontal distance of droplets can lead to the formation of upward layers, thereby reducing the diffusion area of individual droplets [85]. In addition, high-speed droplets colliding with rigid walls can produce sustained reverberation and oscillation behavior similar to the water hammer effect, changing the proportional relationship between contact angle and surface curvature [86,87]. The current control logic rarely takes into account the degradation of settlement performance caused by flow rate fluctuations. Future optimization directions must go beyond single valve control and integrate microscale droplet collision and splashing mechanisms with macroscale pipeline pressure compensation algorithms.

### 3.2. Multi-Actuator Coordination for 3D Crop Spraying

#### 3.2.1. Spatiotemporal Coordination of Proportional Valves and Air Assistance

For the complex canopy of 3D crops, the pesticide application system needs to promote the deep penetration of the spray into multiple layers of branches and leaves. Therefore, the joint modulation of the proportional control valve and the air delivery system becomes the core architecture. Compared to the high-frequency PWM of 2D spray bars, the proportional valve cooperative mechanism can provide a smoother flow response and effectively avoid transient hydraulic shock. Karadol et al. evaluated the performance of a fast-response proportional valve and found that its absolute percentage average application error remained at an extremely low level under constantly changing tractor speeds [88]. The micro herbicide spraying robot developed by Hu et al. utilizes algorithms to optimize multi-nozzle allocation and achieves sub-centimeter-level lateral spraying errors on rough soil surfaces [89].

However, the mechanical hysteresis effect commonly present in proportional control valves limits their performance in high-frequency variable speed operations in orchards. In 3D air-assisted spraying, the more serious challenge is the spatiotemporal coordination delay between the auxiliary air flow and the spray flow. Kramm et al. comprehensively characterized the industrial dual-fluid nozzle, and the experimental data showed that the spray cone angle and single droplet size were most significantly affected by spray pressure [90]. The aerodynamic droplet atomization model proposed by Jackiw et al. provides accurate predictions for spatial resolution distribution in two-fluid atomization by analyzing the formation and rupture mechanisms of ligaments and bag-like structures [91]. The inertia variation in fan speed often lags behind the opening switching of the liquid proportional valve, resulting in a mismatch between the aerodynamic flow field intensity and the liquid injection momentum. How to optimize the matching logic of dual actuators and reduce the action lag caused by mechanical structure while improving the smoothness of adjustment is the key to further improving the accuracy of such technology.

#### 3.2.2. Limitations of Closed-Loop Control and the Faraday Cage Effect

To improve the response time and robustness of multi-actuator collaboration, closed-loop proportional–integral–derivative (PID) control and Programmable Logic Controller (PLC) frameworks have been widely deployed in low-level flow regulation systems. The average spray coverage under different application rates of the dual-side vertical cantilever spray modified by Nauman et al. reached the ideal standard under the adjustment of the microcontroller [92]. Schutz et al. proposed a Generalized Predictive Control (GPC) strategy based on fuzzy gain scheduling to deal with the dead-zone nonlinearity in agricultural spray and ensure the stability of the system under limited parameter changes [93]. In the study of microscale atomization characteristics, Sun et al. demonstrated through experiments that millimeter-scale water droplets hitting the superhydrophobic grid can lead to the collapse of the symmetrical air chamber, and used the inertia-induced hydrodynamic pressure to generate secondary atomization with uniform size [94]. Electrostatic induction-assisted technology combined with specific fluid pressure and spraying height can significantly generate extremely fine droplets and increase the maximum width of charged spray [95].

Research has shown that introducing advanced methods such as Linear Active Disturbance Rejection Control (LADRC) can more effectively shorten system response time and reduce steady-state errors compared to traditional control frameworks [96]. The two-stage Laval tube low-frequency ultrasonic electrostatic atomizer designed by Gao et al. based on fluid mechanics theory can stably output extremely fine ultrafine droplets under the coordinated control of pressure and electrostatic voltage [97]. However, it must be pointed out that electrostatic-assisted technology is physically limited by the Faraday cage effect when facing 3D high-density canopies, and charged droplets are easily captured by the outer blades, resulting in insufficient deposition dose in the inner chamber. The increase in algorithm complexity places higher demands on the processing capability of controllers, and a single feedback mechanism often cannot effectively suppress irregular external fluctuations in complex agricultural environments.

#### 3.2.3. Predictive Control Strategies and Field Dynamic Disturbance Suppression

Advanced predictive algorithms demonstrate superior performance in handling complex farmland disturbances and system nonlinear hysteresis compared to traditional closed-loop control. Koc et al. developed a variable spray prototype equipped with the Internet of Things technology, which achieved a very high coating rate at a specific speed and under electrostatic loading [98]. The electrostatic spray method, based on the bidirectional charging of cotton leaves and pesticide droplets, significantly improves the adhesion of droplets and reduces pesticide residues compared with unidirectional charging [99].

The introduction of LADRC and GPCl strategies marks the transition of flow regulation from passive response to active disturbance suppression. These algorithms have shown significant advantages in overcoming system nonlinearity and time lag, especially in the coordination of multi-electrode electrostatic nozzles, which can effectively improve the spreading rate and rebound height ratio of the liquid on hydrophobic blades [100,101]. Tian et al. analyzed the evaporation characteristics of highly volatile charged droplets and pointed out that corona wind under specific electric fields can enhance mass transfer and significantly shorten the droplet lifespan [102]. The experimental electrostatic charging system developed by Jyoti et al. achieved the optimal mass-to-charge ratio and maximum deposition within the plant canopy at specific electrode voltages and forward speeds [103]. However, in practical embedded agricultural systems, the high computational burden of such algorithms has become a key bottleneck restricting their large-scale field applications. Balancing control accuracy and computational complexity and designing lightweight nonlinear decoupling logic is a necessary path for advanced algorithms to move towards normalized operations. The differences in regulation mechanisms and atomization performance between 2D and 3D variable-rate actuation technologies are compared in Table 3.

### 3.3. Field-Level Spray Control and Dynamic Error Compensation

#### 3.3.1. Interleaved PWM Driving Topology

**Figure 5:**
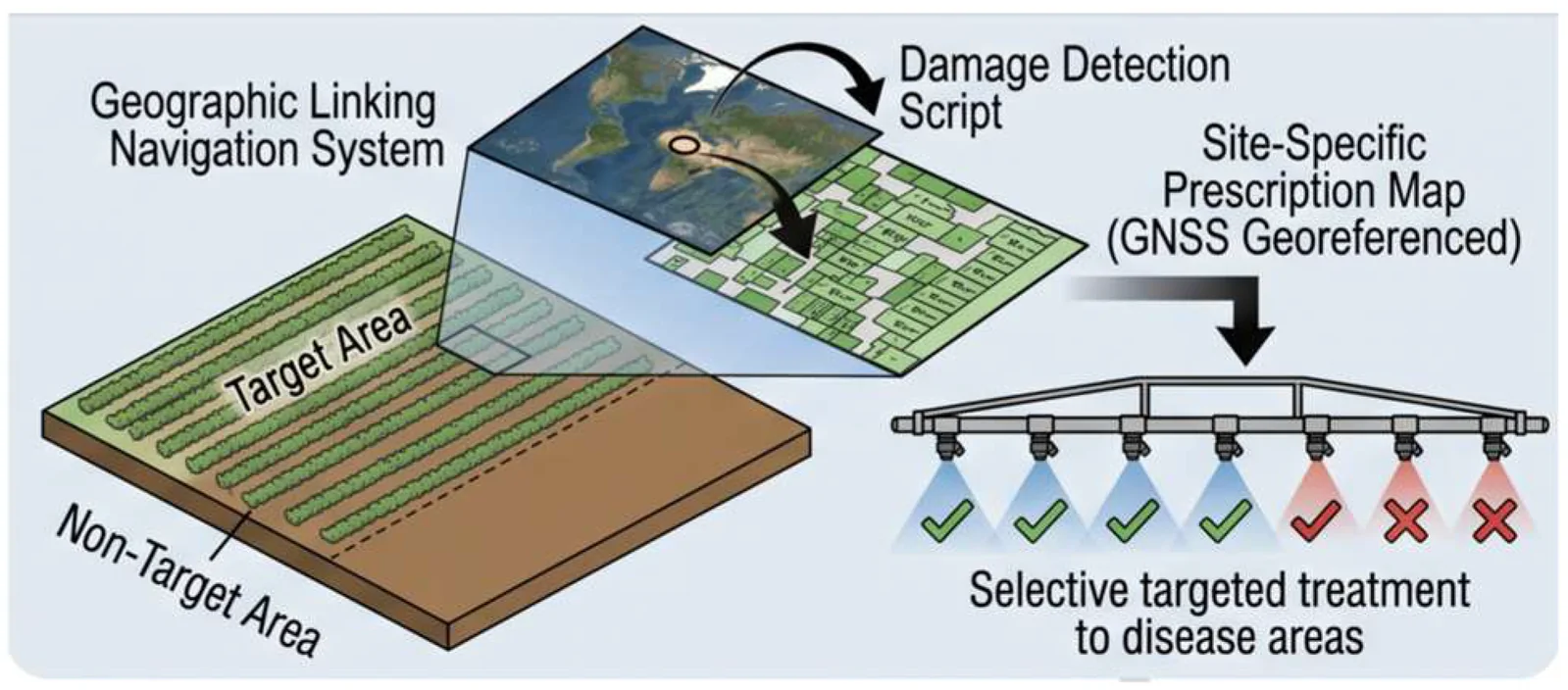
GNSS boundary matching for targeted variable-rate pesticide application.

At the macroscale, pesticide application operations must address the issue of spray deposition caused by dynamic fluctuations in the system. The interleaved PWM driving control method of the low-frequency PWM system proposed by Zhang et al. significantly reduces the pressure fluctuation amplitude upstream of the nozzle by staggering the opening sequence of adjacent nozzles, thus reducing the coefficient of variation in droplet size and effectively eliminating the space distribution discretization caused by intermittent spray [110]. Fluid modeling and microscopic photography research further pointed out that reducing the spray pressure will slow down the reduction rate of droplet size, while adding oil-based detergent of a specific concentration can promote the droplet to change from rebound state to adhesive state, significantly expanding its maximum spreading diameter on the hydrophilic surface [111,112]. Xu et al. established a CFD model to predict the dynamic collision behavior of droplets on rice leaves, and pointed out that droplets with smaller size and lower velocity are more likely to remain on hydrophobic targets [113]. Pandurangan et al. studied the characteristics of droplet clusters in evaporative spray and found that rapid evaporation of droplets would promote their preferential accumulation, thus forming strong spatial concentration heterogeneity [114]. Yuan et al. conducted multiple linear regression analysis on the atomization characteristics of a hollow conical nozzle, and built a volume median diameter prediction model covering three-dimensional spatial variables and spray pressure [115]. Staggered phase drive topology provides a solid engineering support for improving the deposition uniformity of variable spray, but this kind of method requires high phase synchronization accuracy between multiple drive units, and the system wiring complexity increases dramatically. The global navigation satellite system (GNSS)-based boundary-aware control for targeted variable-rate spraying is demonstrated in Figure 5.

#### 3.3.2. Breakpoint Compensation in Autonomous Spraying

During the intermittent operation of large automatic navigation sprayers, the physical hysteresis of the control system often leads to serious leakage and re-spraying. The breakpoint continuous spray system designed by Li et al. based on the lag compensation algorithm, combined with real-time dynamic satellite navigation and wheel odometer, strictly controlled the maximum repeated spraying distance within a very small range and fundamentally eliminated the leakage phenomenon [116]. Zhang et al. discussed the droplet generation process when the upward spray impinges on the downward solid surface, and found that the size of the falling droplet is proportional to the wavelength of the most unstable wave [117].

Research on microscale collision dynamics reveals that the generation process and rebound behavior of droplets when they collide with solid surfaces are influenced by structured surfaces and impurities. The minimum rebound velocity threshold of microscale droplets is significantly increased, and viscous dissipation becomes the main energy loss mechanism [118,119]. Gong et al. focused on the evolution process of perforation in oil-based lotion sheets formed by a plane fan nozzle, and confirmed that the tear mechanism of perforation leads to more extensive early fracture than the puncture mechanism [120,121]. The team’s further experiments showed that a certain proportion of bubble volume would induce the central jet to break, and a specific concentration of lotion could effectively lubricate the hydrophobic surface and promote the final adhesion of hollow droplets [122]. The core of the breakpoint compensation algorithm is to solve the logical lag in automatic navigation and ensure the continuity of the operating trajectory. The current lag compensation technology has been able to reduce the overlapping distance of spray to the centimeter level, but the existing model is highly dependent on the ideal driving path of the vehicle, lacking real-time correction of sudden physical interference such as farmland skidding and chassis pitching.

#### 3.3.3. GNSS-Based Boundary Matching for Independent Nozzle Control

High-precision single-nozzle array control relies on real-time position compensation provided by global navigation satellite systems. Henderson et al. utilized a navigation system with geographic links in conjunction with damage detection scripts to provide precise targeted treatment to disease areas, significantly reducing the treatment area while ensuring effective prevention and control [123]. After the geographical reference prescription map generated by computer vision is imported into the intelligent spray system, the real-time chemical flow is modulated through navigation guidance, which can significantly reduce the use of chemicals while maintaining crop yield [124]. The contact-type electrostatic spray boom system designed by Sun et al. achieved efficient large-area droplet deposition in soybean fields under specific voltage and driving speed [125].

Fluid dynamics simulations and wind tunnel experiments have confirmed that the mechanical vibration of fruit tree leaves caused by airflow generates relative velocity inertial forces, which can improve the impact deposition of low-speed small droplets on dynamic blades [126]. Gong et al. used high-speed microscopic imaging to confirm that the use of oil-based lotion reduces the liquid film area and inhibits the formation of flapping morphology, making the spray speed significantly higher than that of water spray [127]. Xi et al. mathematically modeled the settling behavior of pear tree leaves under wind-induced vibration and pointed out that as long as the droplet velocity and acceleration are within specific thresholds, stable spreading and settling can be achieved on hydrophilic leaves [128]. The spatial geometric matching technology of independent nozzle array improves the spatial resolution of precise pesticide application to sub-meter level, logically avoiding the drift of the spray liquid caused by vehicle trajectory deviation. The focus of current engineering implementation has been upgraded from basic targeted start–stop control to fine geometric boundary matching for complex-shaped crop canopies. Future field compensation algorithms must introduce six degrees of freedom kinematic constraints in three-dimensional space to seek the optimal engineering balance point between macroscopic operating speed and microscopic atomization quality. Table 4 comprehensively summarizes the performance of various low-level control strategies in spray regulation and droplet spectrum control.

## 4. Integrated Platforms and Field Application Scenarios

### 4.1. Unmanned Aerial Spraying Systems and Aerial Applications

#### 4.1.1. Rotor Downwash Flow Field Dynamics and Aerodynamic Droplet Transport

The downwash airflow of crop protection UAVs exhibits unique aerodynamic advantages in promoting droplet penetration and suppressing attenuation. Sanchez Fernandez et al. compared drones with traditional fogging machines and found that drones require less than half the distance to settle 90% of the spray volume [142]. Regarding the physical characterization of downwash airflow, Zhang et al. built a wind field testing platform and revealed the asymmetry of downwash distribution, recording a maximum wind speed of 11.37 m/s within a radial distance of 3.0 m [143]. Although the downwash gas flow has expanded the deposition range, Shi et al.’s 3D simulation shows that this will lead to the expansion of the spray field width to 12.8 m, but the uniformity of the droplet deposition concentration will deteriorate, with fluctuations ranging from 0 to 500 micrograms per square centimeter [144]. To improve distribution uniformity, Lin et al. introduced a waist-shaped charging device to achieve electrostatic activation, resulting in an average coverage rate of 28.4%, which is 10.9% higher than the uncharged state [145]. In terms of aerodynamic parameter monitoring, Huang et al. pointed out that laser trackers can obtain more accurate instantaneous flight trajectories and velocities than airborne sensors, providing more reliable data support for dynamic control of flow fields [146].

The downwash flow field of UAV rotors has great potential in enhancing the penetration of target canopies, but existing research has shown that the asymmetric distribution of downwash airflow can easily lead to severe uneven spatial deposition of fog droplets. Although electrostatic charging technology can improve coverage to a certain extent, the coupling mechanism of gas–liquid–solid multiphase flow under natural crosswind interference still lacks in-depth analysis. Most current simulation models are based on static hovering or ideal constant-speed flight, and fail to fully reflect the real-time damage of complex gust environments to aerodynamic transmission trajectories, which limits the field reliability of adaptive adjustment technology for aerial spraying parameters.

#### 4.1.2. Liquid Tank Sloshing Forces and Structural Stabilization Solutions

During high-maneuverability operations of UAVs, liquid sloshing in the spray tank directly threatens the stability of flight attitude and spray quality. The load variation in the spray tank not only affects the center of gravity, but Pachuta et al. found that it also changes the actual weight and propeller speed of the multirotor drone, thereby affecting the quality of droplet deposition inside the spruce canopy layer [147]. To optimize dynamic operations, Biglia et al. evaluated different flight modes in vineyards and found that strip spraying mode increased canopy deposition by 309% compared to broadcast spraying mode, while reducing ground loss by 54% [148]. The research of da Cunha et al. on coffee crops shows that compared with the standard nozzle, the use of an air induction nozzle with a high spray volume of 20 L per hectare can significantly increase the droplet density and coverage [149]. In addition, Li et al. designed a new two-stage induction electrostatic spraying device, which achieved a peak charge-to-mass ratio of 1.62 mC/kg at a voltage of 12 kV, reducing the median diameter of mist droplets by 23.6% and effectively improving the uniformity of atomization under aerodynamic transmission [150]. Nanavati et al. developed a data-driven optimal path planning framework that minimizes the non-uniformity of coverage distribution by adjusting flight speed and channel width [151].

The current research on the shaking of liquid in spray tanks and the stability of the overall structure mainly relies on the passive weakening of physical disturbances through mechanical baffle design or route planning. However, the continuous impact mechanism of the instantaneous center of gravity shift caused by liquid level shaking on the bottom attitude control system of the aircraft has not been fully revealed. Most existing path planning models are based on ideal static spray widths, ignoring the secondary interference of rotor dynamic compensation on the downwash flow field distribution. Future research needs to directly introduce hydrodynamic feedback from the spray tank into the real-time trajectory correction algorithm of the aircraft to achieve a deeper decoupling of mechanical structure stability and aerodynamic spray distribution.

#### 4.1.3. Path-Planning and Dynamic Obstacle Avoidance in Unstructured Farmland

Path planning and dynamic obstacle avoidance in unstructured farmland are the core of achieving complete autonomous operation of drones. Ahmed et al. reviewed obstacle avoidance methods for agricultural spraying drones and pointed out that spraying drones face dynamic operational and load conditions, heavily relying on data-intensive algorithms to process real-time images [152]. The team subsequently proposed a dynamic obstacle avoidance architecture based on onboard sensor fusion, which successfully avoided all vertical obstacles while maintaining a spray coverage rate of over 98% [153]. Vazquez et al. developed a coverage path planning method for low-altitude work environments, which avoids potential collisions by limiting flight trajectories within the target area [154]. In terms of macro resource utilization and path application, Sahni et al. demonstrated that drones can save 95% of water in maize foliar spraying, and their agronomic performance is comparable to that of tractor spray guns [155]. Rajesh et al.’s study also showed that when the drone flies at a speed of 3 m/s on peanut crops, the target area fog droplet density reaches 31 to 76 droplets per square centimeter, verifying its efficiency under different planned paths [156].

Plant protection drones have a high level of computational maturity in spatial path planning and geometric obstacle avoidance, and innovative electromechanical devices such as side spraying have effectively alleviated the problem of local missed spraying in obstacle avoidance segments. However, current algorithms mostly focus on physical collision avoidance in spatial locations, lacking consideration for the sudden changes in atomization characteristics caused by severe acceleration and deceleration during obstacle avoidance maneuvers. Emergency stop and rapid rotation not only cause severe shaking of the liquid in the spray tank, but also instantly change the angle of the downwash airflow of the rotor, resulting in severe drift of the medicine solution. Integrating spray hydrodynamic constraints directly into multi-objective obstacle avoidance trajectory planning will be a key breakthrough to improve the quality of comprehensive operations.

### 4.2. Autonomous Ground Vehicles (UGV) and Smart Mechanical Interventions

#### 4.2.1. Automated Ground Sprayers (ASV) and Active Boom Suspension Systems

The introduction of automatic ground spray and active suspension systems has significantly improved spraying accuracy in complex terrain. Cui et al. optimized the pendulum suspension parameters using the optimal Latin hypercube design to address the overlapping and leakage of medication caused by the rolling of the spray bar. The root mean square of the spray boom rolling angle was reduced by 14.76%, and the standard deviation was as low as 0.6382° [157]. Li et al. also developed a feedforward proportional– integral–derivative control spray boom active adjustment system, which controlled the spray boom inclination angle at 0.453° when the maximum rolling angle of the chassis was 3.896°, meeting the on-site operation requirements [158]. In terms of front-end perception and decision control, Cakmak et al. designed an automatic orchard ground vehicle equipped with an oscillating arm system, achieving a coverage rate of 60.43%, which was significantly improved compared to the fixed arm system’s 14.87% [159]. Ma et al. conducted in-depth research on the force and motion behavior of leaves during the spraying process and found that under the impact of droplet clusters, the leaves exhibited a downward vibration from 2.58 to 7.75 hertz, with a maximum motion speed of 0.93 m/s. This provides a micro dynamic reference for adjusting the forward speed of the spraying vehicle [160].

The active spray boom suspension system has made significant progress in solving terrain vibration interference, effectively suppressing the damage of mechanical rolling to spray uniformity. However, the direct interaction between chassis stability control and fluid dynamics at the nozzle is still underexplored. The reverse acceleration generated by the suspension during rapid attitude compensation can easily cause transient hydraulic fluctuations inside the pipeline, thereby changing the droplet size spectrum. The current research focuses on isolating the chassis and fluid as two independent physical domains for control, lacking a global collaborative algorithm that integrates the kinematic state of the chassis and the fluid dynamics characteristics of the pipeline, which remains a pain point in high-speed operation scenarios.

#### 4.2.2. Mechanical Canopy-Opening Devices for Dense Crop Canopy Penetration

The high-density crop canopy severely hinders the penetration of medicinal liquids into the lower and middle parts of the plant, and mechanical canopy opening devices and auxiliary airflow have become effective intervention methods to break through this bottleneck. Wu et al. evaluated the mechanical interaction between canopy opener and rice stem through transient dynamics analysis, providing a time displacement mathematical relationship for the optimization of equipment placement [161]. In addition to purely mechanical means, airflow-assisted systems are also widely used to disturb the canopy. Kang et al. evaluated the unmanned ground spray equipped with a pan-tilt nozzle and established a mathematical model to determine the optimal swing angle based on the geometric parameters of the vineyard [162]. Dai et al. tested the low-flow air-assisted spray and determined that the best working condition was 0.65 m/s vehicle speed and 20° nozzle array angle, and the near-end canopy coverage reached 23.67% [163]. Miao et al. designed a powerful air-assisted spray for tall corn crops. The wind speed near the ground exceeded 5 m/s, which expanded the peak deposition area of the lower canopy to 9 to 33 m [164]. Itmec et al. developed an orchard spray that can control the ejected airflow and pesticide spray in real time according to the canopy characteristics, reducing the ground and air drift by 86.95% and 89.98% respectively [165]. Wu et al. pointed out that when strawberry leaves are impacted by critical airflow velocities above 8.7 m/s, high-frequency and high-amplitude motion can significantly improve the penetration and deposition of smaller diameter droplets [166].

The strategy of forcibly changing the canopy structure to promote droplet penetration, whether through mechanical pushing or airflow disturbance, has a significant effect on improving the coverage of the lower layer. However, hard physical collisions can easily cause irreversible mechanical damage to crop stems, while strong wind assistance is accompanied by extremely high energy consumption and intensified drift of small droplets. The existing crown expansion parameters rely heavily on empirical tuning and fail to establish dynamic feedback with the changing biomechanical properties of crops. Future research and development need to delve into the critical equilibrium mechanism between airflow velocity, mechanical thrust, and plant flexible response, in order to seek more flexible and energy-efficient intelligent turbulence actuators.

#### 4.2.3. Precision Micro-Jet Actuators and Targeted Chemical Thinning Systems

The combination of μm-level jet actuators and targeted recognition technology provides a high-precision intervention method for reducing the abuse of chemical agents. Arakawa et al. evaluated the effectiveness of ultra-low capacity pesticide application using drones in chestnut forests and confirmed that drone-targeted spraying, which reduces pesticide use by half, is more effective than traditional methods [167]. Gatkal et al. optimized the operational parameters of UAVs on pigeon pea crops, achieving a thrips control efficiency of 92.45% and 90.12% respectively at a flight height of 1.5 m and a speed of 2 m/s in the top and middle of the canopy [168]. Kumar et al. optimized the cotton field spraying system using response surface methodology and a hybrid GWO-ANN model. At a discharge rate of 3.3 m/s and 2.0 L per minute, the sedimentation coverage reached 9.27%, and the prediction model showed a high coefficient of determination of 0.878 [169]. Safaeinezhad et al. comprehensively compared the UAV with the boom sprayer and found that although the boom sprayer has the highest deposition rate at low speed, the UAV spray quality index is significantly better, and its highly targeted actuator distribution is more suitable for fine operation [170].

Micro spray actuators refine the spatial resolution of agricultural spraying to the level of single flowers or leaves, fundamentally changing the resource waste phenomenon of extensive pesticide application. However, precise microfluidic channels are extremely sensitive to the working environment, and high concentrations of dust, mechanical vibrations, and drug impurities in farmland can easily cause wear or blockage of micro nozzles. In addition, millisecond-level targeted response under high-speed motion is extremely challenging. The current system often performs well in controlled, low-speed evaluations. In the face of complex road conditions and high-throughput operation requirements in actual fields, how to improve the mechanical robustness of microchannel components and simplify on-site maintenance is an engineering gap that urgently needs to be crossed for the large-scale commercialization of such technologies.

### 4.3. Crop-Specific Prescriptions and Multi-Environment Spraying Paradigms

#### 4.3.1. Orchard and Vineyard TRV and LWA Models

For orchards and vineyards with complex three-dimensional structures, TRV and LWA models are the basis for developing prescription diagrams. In terms of micro sedimentation prediction, Liu et al. used binocular cameras and Gaussian process regression to construct a citrus crown sedimentation prediction model, and found that a uniform coefficient of determination higher than 0.79 could be achieved in an area with a density of 4.35 square meters per cubic meter for fitting prediction [171]. Song et al. verified the interaction between wind speed and spray distance on deposition through orthogonal experiments, and provided a quantitative response surface model for dynamic adjustment of fan speed according to different canopy areas [172]. The variable spraying model based on TRV and LWA provides scientific macroscopic dose allocation guidance for the three-dimensional canopy, significantly reducing excessive pesticide application. However, these geometric models are essentially static spatial volume calculations and fail to cover the non-uniform distribution of canopy leaf density during different phenological periods and dynamic interference from environmental wind fields. Especially in sparse canopy applications, simple volume matching can easily lead to drug penetration drift. The future model should evolve from simple 2D or three-dimensional contour fitting to a four-dimensional spatiotemporal dynamic prescription model that includes leaf porosity, branching permeability, and real-time micro-meteorological data.

#### 4.3.2. Controlled-Environment Greenhouse and Aeroponics Nutrient Misting Management

Greenhouse and aerosol cultivation environments have highly controllable characteristics and extremely strict requirements for the microenvironmental distribution of nutrient solutions and pesticides. Guo et al. designed a vertical spray pole electrostatic spray to solve the problem of low prevention efficiency caused by high-density planting of tomato crops in a solar greenhouse, and achieved the best comprehensive leaf coverage under the target distance of 10 kV electrostatic, 0.7 MPa and 35 cm [173]. Lin et al. developed an automatic air-assisted spray based on a single suspension track and evaluated the static and dynamic spray distribution of greenhouse cucumber crops. The axial and radial coverage change coefficients reached 37.2% and 58.3% respectively, confirming the strong influence of moving direction on the distribution [174]. Further research has confirmed that the interaction between specific atomizers and spraying intervals can significantly enhance the photosynthetic efficiency and nutritional value of crops [175]. Zhang et al. applied the EWM-TOPSIS method to regulate greenhouse micro-spraying systems to alleviate high-temperature stress, and pointed out that when the environmental temperature is between 32.5 and 38.6° Celsius, a specific high-frequency application frequency can achieve optimal water use efficiency and thermal toughness [176]. Regarding high-temperature regulation, the combination strategy of micro-spraying and drip irrigation has been proven to reduce leaf temperature by 3 to 6° Celsius above 30° Celsius, maintaining the effective quantum yield of photosystem II [177].

The greenhouse and aerosol cultivation environment are protected from complex meteorological disturbances, providing superior testing conditions for precise control of airflow assistance and electrostatic parameters. However, the enclosed space inside the greenhouse can easily lead to the long-term suspension of tiny droplets after airflow disturbance, causing local high humidity and potential disease growth in non-target areas. In addition, the extremely fine nozzle structure of aerosol cultivation is highly sensitive to salt crystallization in nutrient solution, and the maintenance cost under long-term operation cannot be ignored. The future greenhouse spraying architecture needs to integrate more intelligent space dehumidification systems and anti-scaling nozzle materials to ensure long-term ecological balance in the micro fog environment.

#### 4.3.3. Row-Oriented Strips and Intercropping Target-Oriented Spraying Layouts

For strip planting and intercropping systems, targeted spray layouts can maximize the isolation of pesticide exposure of different crops. Zheng et al. extracted the row navigation line of corn crops and developed an automatic row control system based on the high-clearance sprayer, which achieved an overall inter-row pesticide saving of 11.4% to 20.4% [178]. For large broad-leaved crops such as bananas, Jiang et al. determined that a hollow conical nozzle with a pressure of 0.5 MPa and a wind speed of 3 to 5 m/s can achieve optimal surface wettability distribution and avoid severe leaf deformation [179]. On the deep mechanism of airflow influence, Salas et al. used ultrasonic anemometers to analyze orchard airflow and found that coarse droplets were independent of airflow changes, while fine droplets were highly dependent on airflow conditions, and higher turbulence intensity did not necessarily mean better coverage [180]. Lin et al. proposed an angle correction method based on the principle of cumulative balance of duration by angle, which significantly reduced the difference in left and right canopy cover to −2.3% and 2.9% at a speed of 0.3 m/s [181]. Guo et al. discussed the influence of electrostatic parameters on tomato leaves and concluded that negative voltage combined with falling spray mode can achieve the optimal deposition effect within 2.75 m [182]. Liu et al.’s fluid–structure coupling simulation further confirmed the absolute dominant role of airflow velocity in the distribution of leaf deposition. The higher the velocity, the lower the proportion of leaf tip deposition [183].

The targeted spraying layout for strip and intercropping systems effectively reduces pesticide cross-contamination and improves resource utilization in mixed cropping farmland through physical isolation covers and directional nozzles. However, the current isolation mechanism still appears rigid in dealing with border areas. There are often significant differences in plant height and canopy morphology between intercropped crops, and strong crosswinds can easily penetrate the physical boundaries of the isolation cover, causing herbicides to drift onto sensitive associated crops. Future research on intercropping fields should focus on asymmetric anti-drift wind curtain technology, replacing rigid mechanical shields with flexible airflow barriers to achieve more precise targeted drug blockade in boundary areas. The future intelligent spraying platform should gradually integrate perception, vehicle dynamics, and fluid control into a unified cyber–physical framework, providing the foundation for digital twin-assisted adaptive spraying in unstructured environments.

Table 5 summarizes the performance characteristics of UAV and ground automation platforms in different application scenarios. Through comparison, it can be found that although UAV platforms perform excellently in obstacle avoidance and path flexibility, their system stability is highly susceptible to interference from airflow and load fluctuations. On the other hand, the design of ground platforms focuses on overcoming terrain limitations and canopy sealing issues through physical means such as active suspension and mechanical turbulence. This differentiated layout reflects the complementary nature of current precision spraying technology in the spatial dimension, which achieves comprehensive and refined management from complex three-dimensional canopy surface coverage to internal infiltration through collaborative ground and aerial platforms.

Comparative analysis shows that the integrated spraying platform has great potential in reducing pesticide use and improving efficiency in different crop systems and operational scenarios. However, the performance of these systems is still strongly influenced by coupled multi-physics field interactions, including fluid dynamics, structural vibrations, and aerodynamic disturbances. Although UAV-based side spray systems and ground-based mechanical roof-opening devices effectively address local operational limitations, maintaining consistent spray performance under long-term and highly variable on-site conditions remains challenging. Future research should focus on system-level optimization strategies that combine agronomic prescription models with environmental impact assessment frameworks, in order to more comprehensively evaluate resource efficiency and ecological sustainability.

## 5. Agronomic Performance, Resource Optimization, and Environmental Impacts

### 5.1. Propagation of Perception Errors in Spray Decision-Making and Resource Optimization

In intelligent spraying systems, pesticide reduction and resource utilization efficiency can be regarded as the final task indicators for evaluating the effectiveness of perception and control systems. Existing research shows that fixed-point or variable-rate spraying technologies based on ground perception systems and UAV vision platforms can achieve approximately 30–60% pesticide reduction under different crop types and operational scenarios, including potato fields [192], conventional planting fields [193], sugarcane weed management [194], citrus orchard targeted spraying [195], and apple canopy protection [196]. These technologies have also been validated through modeling studies in tobacco fields [197] and cabbage targeted spraying operations [198]. These results demonstrate that perception-driven variable-rate spraying can further transform target recognition results into spray rate adjustments, thereby achieving optimized resource allocation.

However, existing studies still heavily rely on target recognition accuracy under static or low-speed conditions to evaluate pesticide-saving efficiency, while the mechanism of how perception errors propagate into spray volume calculation and final application performance remains insufficiently understood. During actual field operations, a clear spatiotemporal coupling relationship exists among vehicle motion, sensor sampling frequency, algorithm processing delay, and actuator response time. When the operating speed exceeds 5 km/h, even if visual or deep learning models achieve high recognition accuracy, perception delay and actuator response delay may still cause deviations between the detected target position and the actual spraying location, resulting in perception–execution asynchronization. Therefore, future agronomic performance evaluation should not only focus on the final pesticide reduction rate but also establish an error propagation model covering the entire process from target recognition, spatial localization, spray decision-making, actuation response, to target coverage, incorporating vehicle speed, sensor sampling frequency, and actuator dynamic response into a unified evaluation framework.

### 5.2. Spatiotemporal Response Capability and Dynamic Matching with Operating Speed

Dynamic response capability is an essential feature that distinguishes intelligent precision spraying systems from traditional offline perception and prescription mapping approaches. In practical agricultural operations, the system must complete target perception, data processing, spray rate decision-making, and actuator control within a limited time period. Therefore, reporting only the algorithm inference speed or sensor sampling frequency cannot fully represent the system-level response performance. Existing studies indicate that under high-speed operating conditions, the spatiotemporal delay between vehicle movement and spray execution may lead to positional deviation between targets and spray deposition areas [199,200]. Even with high target recognition accuracy, achieving effective target coverage remains challenging [201].

Therefore, future research needs to establish a spatiotemporal response evaluation framework specifically for precision pesticide application tasks, which should comprehensively consider perception delay, data processing latency, communication delay, actuator response time, and end-to-end system delay. In addition, additional indicators such as spatial delay error and maximum effective operating speed should be introduced to evaluate the effectiveness of perception results during continuous vehicle motion. For UAV spraying systems, the influences of flight speed, flight altitude, rotor downwash airflow, and droplet transport time on perception–execution synchronization should also be considered [202,203].

Therefore, system response evaluation should shift from evaluating only algorithm computational speed to assessing whether the system can execute spraying at the correct position and appropriate time after target displacement. In the future, quantitative relationships among operating speed, sensing distance, processing delay, execution response time, and spatial positioning error should be established, and the maximum effective operating speed of different sensing systems and controllers should be determined accordingly, providing a unified basis for designing responsive and adaptive spraying systems across different platforms [204].

### 5.3. Dynamic Environmental Adaptation for Spray Drift and Off-Target Loss Control

Spray drift and off-target losses are associated not only with nozzle characteristics and droplet properties but also with environmental perception capability and the ability of spraying systems to adapt to dynamic conditions. Existing studies have improved target hit rates to over 64% through genetic algorithm-based vehicle trajectory optimization and dynamic nozzle configuration strategies [205]. In addition, low-cost monocular cameras combined with motion structure algorithms have enabled flow regulation in non-target regions without plant coverage, thereby reducing pesticide losses in canopy gaps [206]. These studies indicate that perception information has gradually evolved from target detection toward active regulation of spray volume and nozzle operating states.

However, transient environmental disturbances, particularly dynamic wind fields, remain major factors contributing to off-target losses. Wind tunnel experiments and particle tracking simulations have demonstrated that when lateral wind speeds vary within the range of 0–6 m/s, the drift potential of conventional hydraulic nozzles exhibits a nonlinear positive correlation with wind speed. The velocity attenuation rate of droplets within 200 mm below the nozzle outlet can reach 50–80% [207,208]. Currently, coarse-droplet anti-drift nozzles, combined with adjuvants such as organosilicon compounds, are widely adopted to reduce the influence of external airflow by increasing the overall droplet size and achieving drift reduction rates exceeding 60% [209].

However, these approaches mainly represent passive disturbance mitigation strategies, where operating parameters are generally predefined before application and cannot be dynamically adjusted according to stochastic gusts and turbulent field conditions. Meanwhile, excessive enlargement of droplet size may effectively reduce drift but can also decrease coverage uniformity and retention capability on complex leaf surfaces, resulting in insufficient deposition within the target area. Therefore, future research should integrate online micro-meteorological sensing, environmental state prediction, and feedforward control mechanisms into intelligent spraying systems. By dynamically adjusting spray angle, flow rate, and droplet size distribution according to wind conditions and canopy characteristics, spraying systems can evolve from fixed anti-drift strategies toward environment-aware active disturbance rejection control.

### 5.4. Collaborative Optimization of 3D Canopy Perception and Spray Deposition

For three-dimensional crops such as orchards, the final performance of intelligent spraying systems depends not only on target recognition capability but also on whether spraying parameters are matched with the spatial characteristics of crop canopies. When high-clearance air-assisted electrostatic spraying systems are applied in vineyards, the electric field can increase the average canopy deposition density by 15.72%, thereby improving effective pesticide deposition within the canopy [210]. For three-dimensional fruit crops such as citrus, optimizing UAV spraying parameters and increasing the application volume from 60 L to 120 L per hectare improved penetration coverage in the middle and lower canopy layers by 52.5%, achieving better disease control performance than traditional backpack spraying methods [211,212]. In high-density cotton canopies, joint regulation of flight height and forward speed can further improve leaf deposition uniformity [213], demonstrating disease interception efficiencies exceeding 75% against typical rice sheath blight [214].

These results indicate that the role of three-dimensional canopy perception extends beyond obtaining geometric information, as it provides essential guidance for determining operational parameters including spray distance, flow rate, flight speed, and droplet transport characteristics. However, electrostatic spraying in dense three-dimensional canopies remains constrained by the Faraday cage effect. Charged droplets are easily captured by outer leaves, causing electric field concentration near the canopy surface and the formation of electrostatic shielding effects. Consequently, droplet penetration into internal canopy regions is restricted, resulting in excessive deposition on external leaves and insufficient pesticide delivery to inner canopy structures. Therefore, simply increasing electric field intensity cannot fundamentally resolve the deposition imbalance within complex canopy environments.

Future intelligent spraying systems should achieve coordinated optimization among three-dimensional canopy perception, environmental condition awareness, and spray execution parameters. For example, LiDAR and depth vision systems can be employed to acquire canopy structural parameters, including height, thickness, and volume, which can then be combined with vehicle speed, spray distance, and local wind field information to dynamically regulate spray volume and spray direction. Such integration would enable closed-loop optimization among canopy structure perception, spray parameter adjustment, droplet transport, and deposition performance. For complex orchard canopies, quantitative relationships between perception errors and deposition deviations across different canopy regions should be established to prevent system evaluation from relying solely on surface point clouds or a single deposition indicator.

## 6. Core Technology Challenges and Future Development Directions

### 6.1. Robust Perception and Intelligent State Understanding in Complex Agricultural Environments

In recent years, the development of multi-source perception technologies, such as machine vision, 3D LiDAR, ultrasound, and active spectral sensing, has provided a data foundation for target recognition, crop structure analysis, and variable-rate pesticide application decision-making in precision spraying systems. However, compared with laboratory or greenhouse environments, actual agricultural production environments exhibit significant uncertainties and dynamic variations. Factors such as natural illumination changes, similarities between soil and crop backgrounds, spatial interference among multiple weed species, and crop canopy occlusion can lead to visual feature degradation and increased target recognition errors. Vibration, attitude variations, and sensor installation deviations generated during field machinery operations can further affect the consistency and reliability of multi-source sensing information.

At present, many studies primarily improve perception accuracy by increasing network depth, expanding training datasets, and optimizing feature extraction algorithms. However, relying solely on increasing algorithm complexity not only increases the computational burden of edge devices but also fails to fundamentally address the problem of incomplete environmental information in agricultural scenarios. For example, relying only on 2D image recognition can determine target locations, but it remains difficult to accurately characterize crop physiological states, canopy spatial structures, and disease or pest severity, which are critical for precise pesticide dosage optimization.

Future perception technologies for intelligent spraying systems need to evolve from single-target recognition to multimodal environmental understanding. By integrating RGB images, 3D point clouds, hyperspectral information, and environmental parameters, heterogeneous sensing data can provide complementary information regarding spatial structures, spectral characteristics, and biological states, thereby improving perception robustness under complex agricultural conditions. Meanwhile, the integration of artificial intelligence approaches, including self-supervised learning, transfer learning, and foundation models, can reduce dependence on large-scale manually annotated agricultural datasets and enhance model generalization across different crop species, growth stages, and geographical regions.

### 6.2. Adaptive Execution and Multi-Physical-Field Collaborative Control in High-Dynamic Spraying Processes

The ultimate objective of precision spraying systems is to transform perception information into stable and accurate pesticide delivery. However, current variable-rate spraying systems still face challenges related to hydraulic dynamic response, actuator delay, and multi-physical-field coupling. In particular, during PWM control, high-frequency solenoid valve switching, and coordinated regulation among multiple nozzles, pipeline pressure fluctuations, liquid transmission delays, and nozzle response delays can cause variations in spray flow rate and droplet size distribution, thereby reducing the spatial consistency of variable-rate spraying.

Existing spray control methods generally consider pumps, valves, and nozzles as relatively independent control units, while neglecting the strong coupling relationships among internal hydraulic pressure transmission, pipeline elasticity, and spray atomization processes. In reality, spraying systems represent typical multi-input multi-output (MIMO) nonlinear dynamic systems, and their execution performance is influenced not only by control algorithms but also by the combined effects of mechanical structures, fluid characteristics, and environmental disturbances. Therefore, adjusting only individual control parameters is insufficient to satisfy the requirements of high-speed and high-precision spraying.

The development of future execution systems should shift from individual component optimization toward system-level collaborative control. At the hardware level, high-response solenoid valves, intelligent proportional valves, pressure regulation mechanisms, and advanced nozzle structures should be further optimized to improve actuator dynamic response and flow regulation accuracy. To address pressure fluctuations and water hammer effects caused by high-frequency PWM regulation, hydraulic structures with pressure buffering and energy dissipation capabilities should be developed to reduce the influence of transient disturbances on spray stability.

Regarding control strategies, fluid dynamic models should be integrated with advanced control theories to achieve dynamic compensation and disturbance rejection during spraying operations. CFD can be employed to analyze internal flow-field variations within spraying systems, and when combined with MPC, active disturbance rejection control (ADRC), and adaptive control methods, the system’s adaptability to pressure variations, vehicle vibration, and environmental disturbances can be improved. For different operation platforms, including UAVs and ground autonomous vehicles, the influence of platform motion states on spraying performance should also be considered to achieve coordinated optimization among flight attitude, vehicle motion, airflow conditions, and spraying parameters.

### 6.3. Cyber–Physical Integration and Autonomous Decision-Making for Closed-Loop Intelligent Spraying

Currently, the development of precision spraying equipment still suffers from the presence of relatively independent modules for perception, mobile platform control, and spraying execution. Although machine vision, variable-rate spraying technologies, and autonomous driving platforms have achieved significant progress, information exchange and collaborative optimization among different modules remain insufficient, limiting the ability of systems to fully exploit real-time environmental information for autonomous decision-making. In particular, effective feedback mechanisms have not yet been established among crop status variations, vehicle motion disturbances, and dynamic changes in spraying processes.

Future intelligent spraying systems should establish a cyber–physical integration architecture for closed-loop control. Among these, CPS should serve as the overall system framework to enable real-time interaction among physical equipment, sensing information, computational models, and control strategies. Digital twin technology can act as an important component within the CPS framework by constructing virtual representations of crop canopy structures, vehicle motion states, spraying dynamics, and environmental conditions, thereby supporting state prediction and decision optimization.

Based on this architecture, the key challenge for future intelligent spraying systems is how to utilize multi-source information to achieve reliable decision-making, rather than merely completing target recognition. Under different crop growth stages and environmental conditions, the system needs to comprehensively consider pest and disease risks, canopy structures, spray deposition performance, environmental drift risks, and economic costs to achieve dynamic optimization of spraying dosage and operational strategies.

Future research should further develop intelligent decision-making algorithms that integrate mechanistic models with data-driven approaches. By combining reinforcement learning, physics-informed neural networks, and model-based optimization methods, researchers can improve the autonomous adaptability of spraying systems under complex agricultural environments. Establishing long-term continuous data feedback mechanisms will further enhance model reliability and cross-regional applicability, promoting the transition of precision spraying equipment from automated execution toward autonomous decision-making.

## 7. Conclusions

### 7.1. Development Status and Main Progress of Intelligent Precision Spraying Technologies

Precision agricultural spraying technology is undergoing a transformation from traditional uniform application approaches toward intelligent spraying paradigms based on multi-source sensing, decision optimization, and variable-rate control. This review systematically summarizes recent advances in perception, decision-making, and execution technologies within the field of precision spraying. In terms of perception, multi-source sensing technologies—including machine vision, 3D LiDAR, ultrasound, and active spectral sensing—have continuously improved crop target recognition, canopy structure characterization, and working environment understanding, providing essential data support for precision spraying operations. In terms of execution, the development of PWM control, variable-rate flow regulation, intelligent valve control, and adaptive control strategies has improved the spatial resolution and dynamic response capability of pesticide delivery, gradually transforming spraying operations from traditional fixed-parameter control toward adaptive and dynamic regulation.

The development of autonomous agricultural platforms, including UAVs and ground autonomous vehicles, has further expanded the application scenarios of precision spraying technologies, enabling operations in complex terrains and three-dimensional crop canopy environments. However, current intelligent spraying systems still face several challenges, including insufficient environmental adaptability, limited reliability in multi-source information fusion, inadequate dynamic response of execution systems, and incomplete integration among perception, decision-making, and execution modules. These limitations remain key challenges that must be addressed to promote the large-scale deployment of intelligent spraying technologies.

### 7.2. Intelligent Closed-Loop Spraying and Future Directions for Sustainable Agricultural Development

The future development of precision spraying systems will not rely solely on improvements in individual sensing technologies or control algorithms; it will require the construction of intelligent closed-loop systems that deeply integrate perception, decision-making, and execution. By employing multimodal sensing technologies to enhance environmental information acquisition, utilizing intelligent decision-making approaches to optimize spraying strategies, and integrating high-performance execution mechanisms to achieve precise agrochemical application, the efficiency of agricultural input utilization can be further improved.

The development of CPS, digital twin technologies, and artificial intelligence methods provides new technological pathways for constructing autonomous and dynamically adaptive spraying equipment. Future intelligent spraying systems should not only ensure effective crop protection but also comprehensively consider resource utilization efficiency, environmental impacts, and economic benefits, thereby achieving a balance between agricultural productivity and ecological sustainability.

Overall, precision spraying technologies are evolving from traditional mechanized operation modes toward a new generation of intelligent systems that integrate robust perception, dynamic decision-making, and autonomous execution. With the continuous reduction in sensor costs, improvements in edge computing capabilities, and advances in intelligent control theories, next-generation spraying equipment is expected to achieve more accurate, efficient, and environmentally sustainable agrochemical management, providing important technological support for the development of future smart agriculture.

## Figures and Tables

**Table 1:** Spatiotemporal and algorithmic comparisons of vision-based sensing paradigms in precision spraying.

Target and Canopy Type	Actuation Equipment	Algorithms and Models	Advantages and Validation Accuracy	Limitations and Field Challenges	Ref.
Weed identification. Row localization. 2D open fields.	Boom sprayer	YOLO variants. VGG networks.	Low edge inference latency. High accuracy in controlled setups.	Poor generalization under variant lighting/soil backgrounds. Severe accuracy degradation at high operational speeds.	[26,27]
Canopy porosity. Depth estimation. 3D orchards.	Air-blast sprayer	Depth mapping. Stereo vision reconstruction.	Cost-effective. Extracts the localized foliage density.	Lateral view susceptible to branch occlusion. Severe depth noise occurs under intense ambient sunlight.	[28,29]
Foliar physicochemical characterization. Microscopic interface.	Smart micro-jet system	Dynamic contact angle models. High-speed camera tracking.	Uncovers retention dynamics. Quantifies interface spreading thresholds.	Difficult real-time parameter extraction at high speeds. Extreme multi-physics decision complexity.	[30,31,32]

**Table 2:** Analysis of 3D stereoscopic crop canopy quantitative active remote sensing technologies.

Sensor Type and Data Fusion	Core Canopy Metrics	Resolution and Error Characteristics	Application Scenarios and Technical Features	Ref.
3D LiDAR + Kinematic RTK	Voxel mapping. TRV.	Tree height RMSE < 0.45 m.	Large-scale digital orchards. High-precision geometry. High hardware costs and computational load.	[47,48,49]
Solid-State LiDAR Module	3D canopy profiling. LWA.	Ultra-low RMSE at close range.	Greenhouses and high-density orchards. Short- to medium-range tracking. Cost-effective.	[50,51,52]
Ultrasonic Multi-Echo Sensor Array	Canopy thickness. Leaf Area Density (LAD).	Static relative error: 1.4–5.4%.	Real-time dynamic feedback. Light and color immunity. Low spatial resolution.	[53,54,55]

**Table 3:** Spatiotemporal and fluid-dynamic comparison between 2D and 3D variable-rate actuation mechanisms.

Actuation Technology	Core Regulation Mechanism	Target Crop and Platform	Droplet Size Spectrum Distortion Evaluation	Ref.
Pulse-Width Modulation (PWM) Solenoid Valves	High-frequency duty cycle modulation for individualized nozzle-level flow control.	2D arable row crops.	Low duty cycles, lower atomization energy, causing droplet coarsening. Pressure spikes during transient switching generate drift-susceptible satellite micro-droplets. High-frequency shutoff induces severe water hammer effects in pipelines.	[104,105,106]
Proportional Control Valves (PCV) & Air-Assisted Systems	Valve orifice area and pump speed adjustment coupled with fan-driven air carriers.	3D tree canopies.	Nozzle liquid sheet breakup remains structurally stable. Excessive air blast velocity introduces severe aerodynamic shear, which over-fragments droplets. Mechanical hysteresis and air–fluid spatiotemporal mismatches induce localized off-target drift.	[107,108,109]

**Table 4:** Analysis of low-level control strategies, transient fluidics, and droplet spectrum distortion behavior.

Control Strategy and Actuation	Target Dimension	Key Transient Fluidic Parameters	DSD Distortion and Phenomenon	Deposition Uniformity and Efficiency Metrics	Ref.
PWM-Based Variable Flow Control	Planar Canopy	Current reduced by 92% Prediction RMSE: 1.96%	Minimal DSD distortion due to optimized coil temperature	Stable flow control based on precise activation time	[129]
Model Predictive Control (MPC)	Planar Canopy	Zero steady-state error Low transient response	Mitigates DSD shifting via stabilized system pressure	Reduced peak overshoot and chemical waste compared to PID	[130]
Output Linearization Adaptive GPC	Volumetric Canopy	Dynamic output compensation Real-time resistance estimation	Prevents DSD shift during high-frequency nozzle switching	Robust tracking accuracy under embedded constraints (STM32)	[131]
RBFNN-SMC Control	Planar Canopy	Overshoot reduced by 11.2% Flow tracking error: 3.2%	Dampens hydraulic oscillation under lag conditions	Steady-state error restricted within 0.51%	[132]
Fuzzy-Adaptive Control via ESO	Volumetric Canopy	Multi-iteration GrabCut inputs Dual flow pressure regulation	Prevents pressure spikes that distort atomization	Spray volume reduced by 59.73%; utilization up by 61.42%	[133]
Aerial Downwash Actuation	Volumetric Canopy	Varying rotor wind field Dependence on solution concentration	Active ingredient carrier dynamics vary with canopy size	Lower physical coverage on large trees; severe ground loss	[134]
UAV-SfM Grid Mapping	Volumetric Canopy	Grid heights: 0.2–10.0 m Spatial width: 5.0 m	Guided parameters prevent unintended micro-droplet shifts	High correlation (R^2^ > 0.97) with LiDAR canopy profiling	[135]
Crossflow Inertial Scaling	Micro Boundary	Crossflow velocities: 1, 3, 5 m/s Ballistic drift modeling (R^2^ > 0.93)	Self-similar DSD normalized by local Dv50 value	Segregation governed by inertia rather than turbulence	[136]
Multimodal Size Metrology	Micro Boundary	Droplets > 1200 μm non-spherical Air bubble entrainment errors	Bubbles bias PDPA data; diffraction overestimates fines	Metrology limitations skew true DSD distortion analysis	[137]
Canopy Segmentation Audit	Volumetric Canopy	Trailer, mounted, hand-held sprayers	Mechanical air-blast shearing alters localized DSD	Trailer sprayer achieves highest deposition	[138]
Fixed Spray Array System	Volumetric Canopy	Swirl nozzles droplet size: 240 μm Installation height: 50–100 cm	Fixed plate sprinklers achieve tighter DSD span	Sprinkler penetration ratio (71.67%) outperforms swirl nozzles	[139]
TRV-Based Dosage Adjustment	Volumetric Canopy	Reduced chemical volume: 75% Seasonal dose saving: 60.7%	Vertical boom arrays match DSD to late-stage canopy	Maintained equal threshold of pest and disease control	[140]
Response Surface Optimization	Volumetric Canopy	Specific volume: 0.077–0.093 L/m^3^ Forward speed: 0.43–0.95 m/s	Flow rate constraints stabilize terminal atomization	Inner canopy zone deposition is consistently lower than outer	[141]

**Table 5:** Comprehensive comparison of the performance and limitations of spraying systems across different integrated operation platforms and field application scenarios.

Platform and Scenario	Core Intervention Strategy	Key Metrics	Ref.
UAV Aerial Aerodynamic Transport	Rotor downwash flow optimization	Droplet velocity decay rate reduced from 36.72% to 13.21%	[184]
UAV High-Maneuverability Flight	Flat hexagonal spray tank with internal anti-sloshing baffles	Mitigated omnidirectional fluid sloshing; maintained trajectory precision	[185]
UAV (Unstructured Obstacle Avoidance)	Auxiliary side-spray nozzles integrated with Lattice Boltzmann wind field simulation	Effective swath width in obstacle neighborhoods increased by 15.25%	[186]
UGV/ASV Rugged Terrain Operation	Electro-hydraulic active suspension with optimal-weight data fusion	Peak boom rolling angle at resonance suppressed from 1.29° to 0.72°	[187]
Boom Sprayer Dense Rice Canopy Penetration	Front-mounted rigid mechanical canopy opener and flow divider	Foliar coverage on paraxial leaf surfaces increased by 72.91% at 1.2 m/s	[188]
Tractor sprayer (Orchard TRV)	2D LiDAR scanning matched with TRV prescription model	Chemical volume reduced by 78%; over-spray reduced by 28%	[189]
Aeroponic greenhouse	11.24 μm air-assisted nozzle system	Stabilized root-zone pH/EC; increased root biomass	[190]
Intercropping system	Anti-drift shields + drop pipe zoning	Deposition: soybean 40–50%, maize 50–68%	[191]

## Data Availability

Data availability is not applicable to this article as no new data were created or analyzed in this study.
